# Conditioned medium from mesenchymal stem cells improves condylar resorption induced by mandibular distraction osteogenesis in a rat model

**DOI:** 10.1016/j.heliyon.2021.e06530

**Published:** 2021-03-16

**Authors:** Wataru Katagiri, Satoshi Endo, Ryoko Takeuchi, Daisuke Suda, Naoaki Saito, Tadaharu Kobayashi

**Affiliations:** Division of Reconstructive Surgery for Oral and Maxillofacial Region, Faculty of Dentistry & Graduate School of Medical and Dental Science, Niigata University, 2-5274 Gakkocho-dori, Chuo-ku, Niigata, 951-8514, Japan

**Keywords:** Condylar resorption, Orthognathic surgery, Mesenchymal stem cells, Secretome, Conditioned media, Regenerative therapy

## Abstract

Condylar resorption (CR) after surgical orthognathic treatment is defined as dysfunctional remodeling of the temporomandibular joint manifested by morphological changes with decreased condylar head volume that cause occlusal and esthetic changes. Although both conservative and surgical treatment strategies have been employed for the treatment of CR, effective procedures have not been established till date. In this study, the effects of MSC-CM on CR were investigated. Bone marrow-derived MSCs of rats (rMSCs) were cultured until 80% confluent, cultured in serum-free conditioned medium for 48 h; the collected medium was defined as MSC-CM. Osteogenesis, chondrogenesis, and angiogenesis-related gene expression in rMSCs cultured with MSC-CM was evaluated by quantitative real-time polymerase chain reaction. A rat CR model was used for animal studies, in which CR occurred after mandibular distraction osteogenesis for 10 days. MSC-CM was injected via the tail vein and quantitative and qualitative evaluations were performed by micro-computed tomography (micro-CT) and histology.

MSC-CM enhanced osteogenesis-, chondrogenesis-, and angiogenesis-related gene expression in rMSCs. Micro-CT showed CR in control groups; however, it was observed to be improved in the MSC-CM group. Histologically, an enlarged cartilage layer was seen in the MSC-CM group, while cartilage layers had almost thinned or disappeared in control groups. These results indicate that MSC-CM improved CR.

## 1 Introduction

Condylar resorption (CR) after surgical orthognathic treatment is defined as dysfunctional remodeling of the temporomandibular joint (TMJ) manifested by morphological changes along with decreased condylar head volume [1, 2]. The pathogenesis of CR is due to imbalance between mechanical stress and the ability to adapt to it in the remodeling of condylar head, leading to shorting of ramus height, retrusion, and overbite with clockwise rotation of the mandible [3, 4, 5]. Although conservative treatment using occlusal splint, orthodontic treatment, and surgical treatment including transplantation of bone and artificial joint replacement have been proposed for the treatment of CR [2, 6, 7], till date, effective procedures have not been proven.

As described above, treatment for CR mainly aims to reduce mechanical stress to TMJ and/or replace the missing condylar bone. However, regenerative medicine has not yet been applied for treatment of CR.

Our previous study, using rat models with mandibular distraction osteogenesis (DO), suggested that continuous overloading of mechanical stress results in CR [8, 9]. Because condylar bone is usually subjected to mechanical stress during jaw movement and mastication, CR is likely to occur when such stress exceeds bone tolerance [8]. Using this model, Suda et al. [9] revealed that FK506, an immunosuppressant which is known to promote osteoclast activity, induced serious CR because administration of FK506 altered bone mass and architecture.

Several studies have reported that mesenchymal stem cells (MSCs) secrete a variety of growth factors and cytokines, and that paracrine effects of the growth factors and cytokines secreted from implanted MSCs may promote tissue repair [10, 11, 12]. The paracrine factors secreted by MSCs can accumulate in conditioned medium during cell culture. Serum-free conditioned medium from human MSCs (MSC-CM) has been reported to serve multiple positive functions [13, 14]. In particular, studies conducted till date have supported the theory that the paracrine factors included in MSC-CM are important for turnover of local bone status [15, 16].

We have reported that MSC-CM contains numerous growth factors such as insulin growth factor-1 (IGF-1), vascular endothelial growth factor (VEGF), and transforming growth factor-β1 (TGF-β1), which accelerate osteoblastic differentiation and bone regeneration [13, 14]. These cytokines present in MSC-CM were revealed to act as effective factors for bone regeneration through the enhancement of endogenous cellular recruitment such as of vascular endothelial cells and stem cells [17]. We also reported that MSC-CM promoted differentiation of osteoclasts and expression of master regulatory transcriptional factors for osteoclastogenesis. In addition, MSC-CM showed functional maintenance in osteoclasts despite the presence of bisphosphonates and RANKL inhibitors [15, 16].

Based on these findings, we hypothesized that MSC-CM improves CR through endothelial cellular recruitment, angiogenesis, and maintenance of remodeling of the condylar tissue.

## 2 Materials and methods

### 2.1 Cell preparation

All the animal experiments undertaken in this study were performed in strict accordance with the protocols that were reviewed by the Institutional Animal Care and Use Committee of Niigata University (approval No. SA00456).

Rat MSCs (rMSCs) were isolated from 7-week-old Wistar/ST male rats (Japan SLC, Shizuoka, Japan) as previously reported [18]. Briefly, donor rats were sacrificed, and femora were dissected out. Under sterile conditions, the edge of each bone was cut, Dulbecco's modified Eagle's medium (DMEM; Gibco, Rockville, MD, USA) was injected into the bone marrow using an 18-gauge syringe, and the bone marrow cells were flushed out from the opposite side; this procedure was repeated several times. Each bone marrow sample was then seeded in a tissue culture flask in DMEM containing an antibiotic–antimycotic solution (100 units/ml penicillin G, 100 mg/ml streptomycin, and 0.25 mg/ml amphotericin B; Gibco), and the medium was supplemented with 10% fetal bovine serum (FBS). Three days after seeding, floating cells were removed, and the medium was replaced with fresh medium. Adherent, spindle-shaped cells were passaged when the cells reached confluence. The adherent cells were collected using trypsin/EDTA, resuspended in fresh medium, and transferred to new flasks at a density of 1×10^4^ cells/cm^2^.

rMSCs obtained from the cultures at the 2nd to 4th passages were used for the experiments. Pluripotency of rMSCs for differentiation into classic mesenchymal lineage cells, including osteoblasts, was verified using the previously reported methods. Briefly, the rMSCs were cultures with MSC-CM or DMEM with 5% FBS for 14 days. After 14 days, rMSCs were washed twice with PBS and fixed with 10% neutral formalin for 15min. The cells were stained with alizarin red S (FUJIFILM Wako Pure Chemical Co., Osaka, Japan) for 30 min. The mineralized depositions were evaluated by light microscopy.

### 2.2 Preparation of conditioned media

The rMSCs that were 80% confluent were re-fed with serum-free DMEM [DMEM(−)]. Conditioned media of the cultured cells were collected after an additional 48 h of incubation and filtered through a 0.22 μm filter sterilizer. The collected cultured conditioned medium was designated as MSC-CM and stored at −80 °C before use.

### 2.3 RNA extraction and quantitative reverse transcriptase-polymerase chain reaction (qRT-PCR)

rMSCs were cultured in DMEM (−) with or without the addition of MSC-CM for 48 h. Total RNA was extracted using RNeasy Mini kit (QIAGEN N.V., Venlo, Netherlands), treated with RNase-free DNase set (QIAGEN) to remove potential genomic DNA contamination, and then reverse transcribed into cDNA using PrimeScript RT Master Mix (TaKaRa Bio Inc., Shiga, Japan) according to the manufacturer's instructions. qRT-PCR analysis was performed using TB Green Premix Ex Taq II (TaKaRa Bio) in combination with Thermal Cycler Dice Real Time System III (TaKaRa Bio). The sequences of specific primers of osteogenesis-related genes [*alkaline phosphatase* (*ALP*), *Runt-related transcription factor 2* (*Runx2*) and *type I alpha1 collagen* (*COL Iα*)], chondrogenesis-related genes [*type II alpha1 collagen* (*COL 2α*), *SRY-box transcription factor 9* (*SOX9*) and *aggrecan* (*ACAN*)], and angiogenesis-related genes [*angiopoietin 1* (*ANG1*) and *vascular endothelial growth factor* (*VEGF*)] are listed in Table 1.

**Table 1:** Primer sequences used for qRT-PCR.

Gene	Sequence	Accession no.
*ALP*		NM_013059.1
F	CATCGCCTATCAGCTAATGCACA	
R	ATGAGGTCCAGGCCATCCAG	
*Runx2*		NM_001278483.1
F	CATGGCCGGGAATGATGAG	
R	TGTGAAGACCGTTATGGTCAAAGTG	
*Col1a1*		NM_053304.1
F	GACATGTTCAGCTTTGTGGACCTC	
R	GGGACCCTTAGGCCATTGTGTA	
*Col2a1*		NM_012929.1
F	GAGGGCAACAACAGCAGGTTCAC	
R	GCCCTATGTCCACACCAAATTC	
*Sox-9*		NM_080403.1
F	CAGGAAGCTGGCAGACCAGT	
R	GGTCTCTTCTCGCTCTCGTTCA	
*Acan*		NM_022190.1
F	TGGCATTGAGGACAGCGAAG	
R	TCCAGTGTGTAGCGTGTGGAAATAG	
*Ang-1*		NM_053546.1
F	GGCGAGTGCTGGCAGTACAA	
R	TGAGTCAGAATGGCAGCGAAG	
*Vegf-a*		NM_031836
F	TCCTGCAGCATAGCAGATGTGA	
R	CCAGGATTTAAACCGGGATTTC	
*GAPDH*		NM_017008.4
F	GGCACAGTCAAGGCTGAGAGAATG	
R	ATGGTGGTGAAGACGCCAGTA	

### 2.4 Rat mandibular distraction osteogenesis

**Figure 1:**
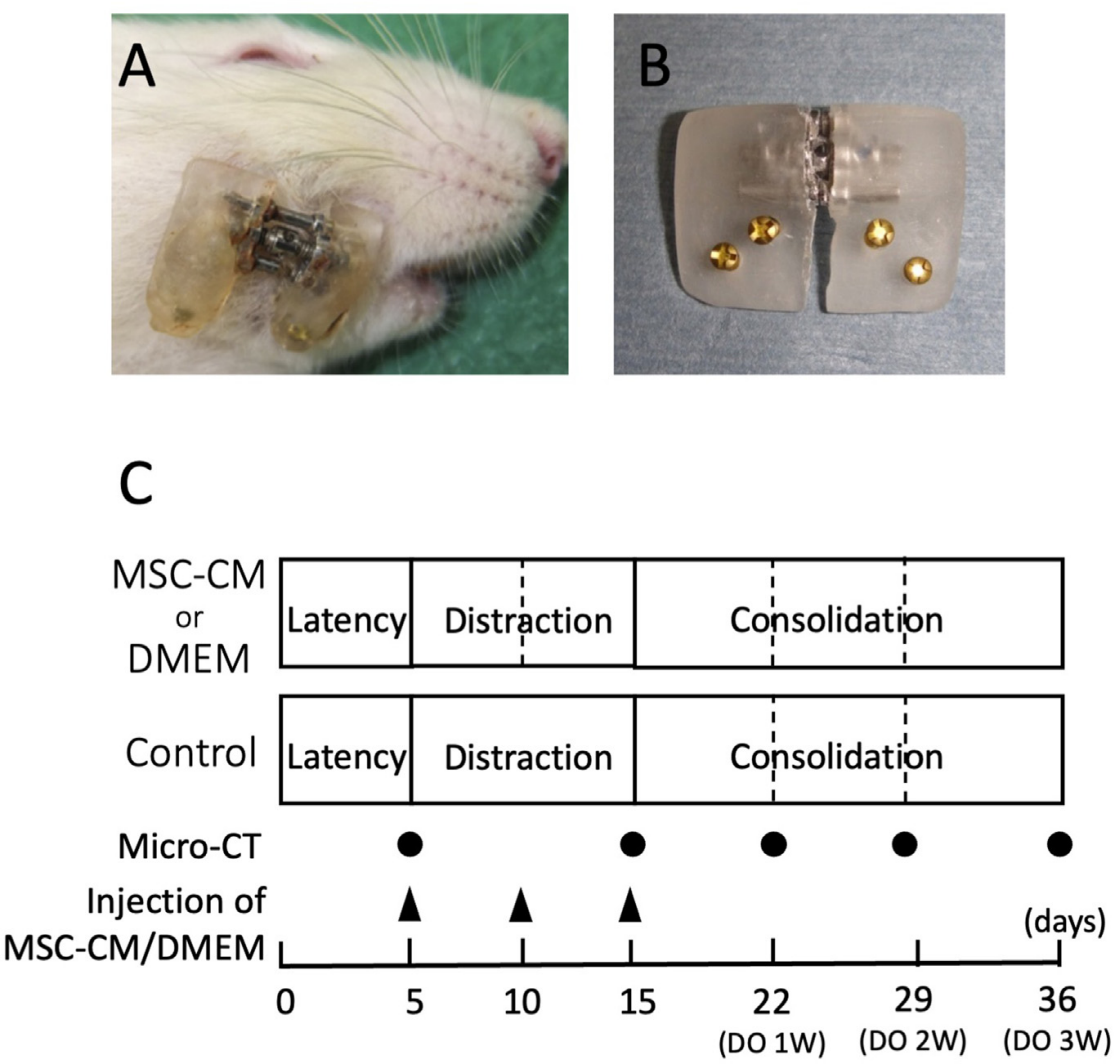
Rat mandible with a distraction device and mandibular distraction protocol of this study. (A) Lateral view of a rat mandible with a distraction device. B) A custom-made distraction device. The components include the body of the device made from acrylic resin, titanium bone screws for fixation of the device, and an expansion screw for active distraction. (C) Mandibular distraction protocol. Black dots indicate the time required for micro-CT scanning. Black triangles indicate the timings of injection of MSC-CM or DMEM.

A distraction device was built using an orthodontic jackscrew (Dentaurum, Ispringen, Germany) with each end embedded in acrylic resin, as previously described [8, 9]. The device was attached to the rat mandible using four self-tapping titanium bone screws (Stryker Leibinger, Freiburg, Germany; Figure 1A, B). Each 90-degree turn of the jackscrew separated the osteotomized bone edges by 0.175 mm. Distraction surgery was performed as described previously [8, 9]. Briefly, under anesthesia with an intraperitoneal injection of 8% chloral hydrate (400 mg/kg) and a local injection of 2% lidocaine, the right hemimandibular bone was exposed. The buccal cortical bone was carefully cut between the second and third molars with a double-sided diamond disk (Horico, Berlin, Germany) and a fissure bur (Shofu, Kyoto, Japan). Two bicortical holes were made, mesial and distal to the cutting line, and two self-tapping titanium bone screws were inserted into these holes. Two additional titanium bone screws were also threaded close to the previous screws to further maintain the stability of the distraction device. The lingual cortical bone was then cut with a fissure bur, and the mandibular bone was fractured. The distraction device was attached to the exposed screws with acrylic resin. Soft tissues and skin were sutured, and Penicillin G (2000 U/100 g body weight/day) was administered intraperitoneally for 5 days after the operation. Figure 1A shows the lateral view of the rat mandible following application of the distraction device.

The distraction protocol included a 5-day latency phase following the surgery. Distraction was gradually performed at a rate of 0.175 mm every 12 h for 10 days, which resulted in a total lengthening of 3.5 mm. The distraction device was maintained at this distance for 21 days during the consolidation phase (Figure 1C).

### 2.5 Micro-CT and bone structural analysis

Specimens from the mandibular bone were scanned and 3-dimensional (3-D) images reconstructed by micro-computed tomography (micro-CT) (Figure 4A, B; CosmoScan GX; Rigaku, Tokyo, Japan) at 90 kV and 88 μA, with a voxel pitch of 50 μm, pixel size of 50 μm, and projection number 500 using Analyze software (Ver.11.0, Rigaku, Tokyo, Japan). CR was evaluated by calculating total bone volume (BV) and total tissue volume (TV). We defined BV as the area consisting of trabecular bone (yellow, Figure 4A) and cortical bone (green, Figure 4A), which represented 450 to 600 CT values and 600 to 2000 CT values, respectively. The CT value in this micro-CT system was calculated by Analyze software and was an approximated value to Hounsfield Units (HU). TV was calculated as BV + other area (blue, Figure 4A), which mainly consisted of bone marrow representing 350 to 450 CT values.

Bone mineral density (BMD) of the condylar bone was evaluated by calculating the BV/TV value using Analyze software. BMD was evaluated within a square of 1000 × 1000 μm of the condylar bone facing the articular tubercle, and three sliced regions were set at 250 μm intervals from each CT data.

### 2.6 Tissue preparation, histological and immunohistochemical evaluation

At the end of the experimental period, the rats were perfused transcardially with 4% paraformaldehyde in 0.1 M phosphate buffer pH 7.4 under deep anesthesia. The mandibular bones and surrounding tissues were then extracted and immersed in the same fixative at 4 °C for 24 h. After micro-CT analysis, samples were decalcified in a 10% ethylenediaminetetraacetic acid disodium solution for 6 weeks at 4 °C and then embedded in paraffin. Serial sagittal sections of the mandibular condyle were prepared at a thickness of 5 mm for histological examination. Sections were dewaxed, rehydrated, stained with hematoxylin-eosin, and analyzed using a light microscope (FX630, OLYMPUS Co., Tokyo, Japan) and using FLVFS-LS software (OLYMPUS Co.).

Immunohistochemical staining was performed for CD44 (1:1000; ab189524, Abcam PLC, Cambridge, UK) to evaluate osteogenesis, while staining for VEGF (1:1000; ab39250, Abcam) was performed to detect angiogenesis. The sections were rehydrated, subjected to antigen retrieval using citrate buffer (pH 6.0) for 10 min at 121 °C, and blocked for endogenous peroxidase with 0.3% H_2_O_2_ in methanol and incubated for 30 min. After washing with PBS, the sections were blocked for non-specific binding using 5% skim milk solution for 1 h, and then incubated with the primary antibody overnight at 4 °C. Subsequently, the sections were reacted with EnVision Plus (Dako, CA, USA) for 1 h and developed with 3,3-Diaminobenzidine (DAB) solution. Hematoxylin counterstaining was performed following the DAB reaction.

### 2.7 Statistical analysis

All data were analyzed as the mean ± standard deviation (SD). Comparisons between experimental groups and control groups were performed using Tukey's honestly significant difference test. Differences were considered statistically significant at *p* < 0.05.

## 3 Results

### 3.1 Morphology of rat MSCs and quantification of MSC-CM

**Figure 2:**
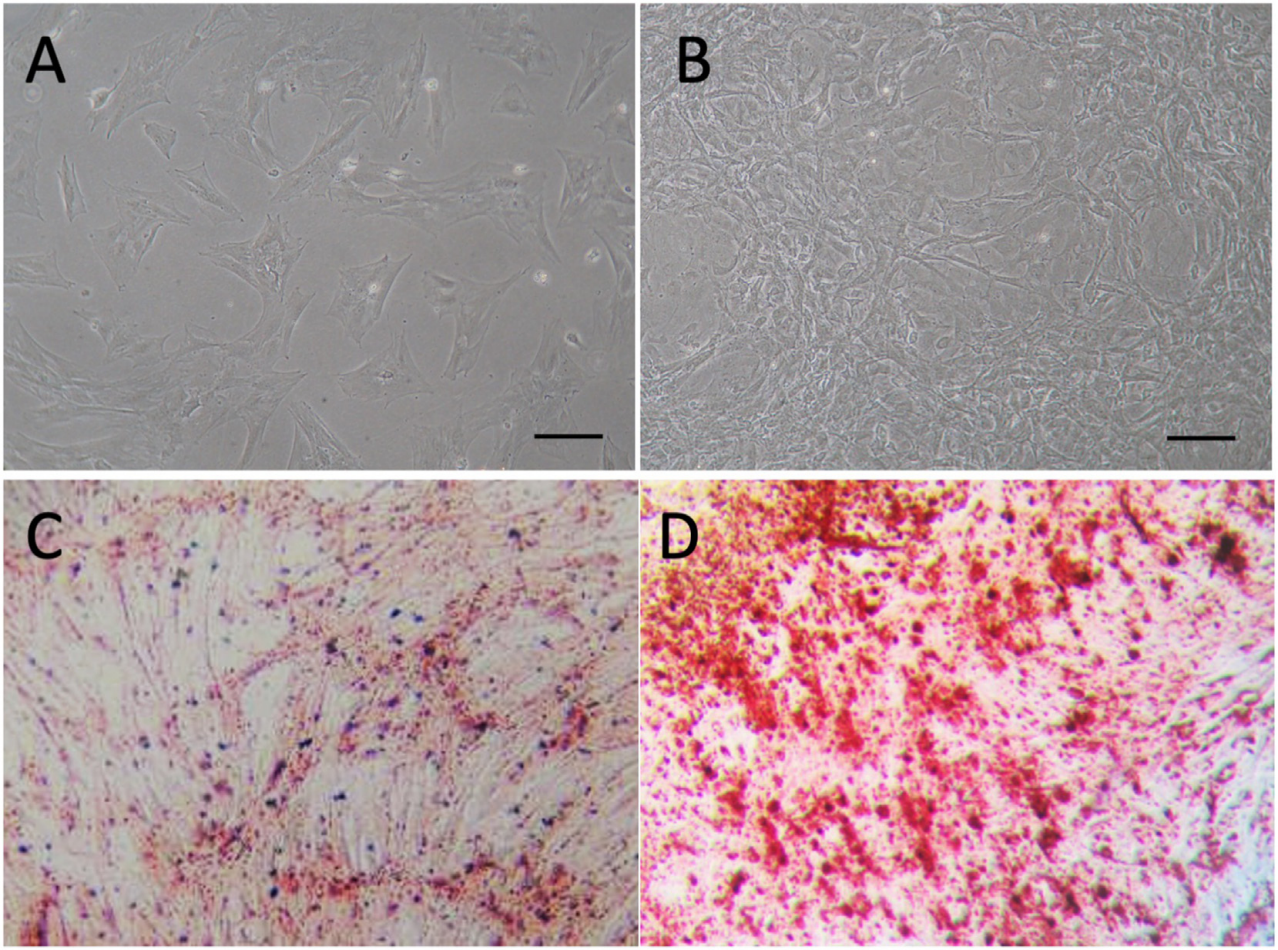
Morphology of rat MSCs and quantification of MSC-CM. (A, B) Phase contrast images of rMSCs showed spindle shaped, fibroblast-like morphology. rMSCs cultured for 3 days (A) and for 7days (B). Bar = 200μm. (C, D) Mineral depositions by Alizarin red S staining. rMSCs cultured with DMEM(-) showed no mineral depositions (C), however many depositions were observed in rMSCs cultured with osteogenic medium (D) (x40).

Phase contrast images of the cultured cells obtained from the rat bone marrow in this study showed that these cells were spindle shaped, fibroblast-like morphology (Figure 2A, B). Mineral depositions by Alizarin red S staining were observed in rMSCs cultured with DMEM-10% FBS (Figure 2C) or the osteogenic induction medium (Osteogenic Differentiation Medium BulletKit™ (Lonza, MD, USA) for 14 days (Figure 2D). These findings suggested that these cells had characteristics of MSCs.

MSC-CM used in this study was quantified by the concentration of growth factors including IGF-1, TGF-β and VEGF by enzyme-linked immuno sorbent assay (ELISA) (data not shown).

### 3.2 MSC-CM regulates expression of osteogenic, angiogenic, and chondrogenic marker genes

**Figure 3:**
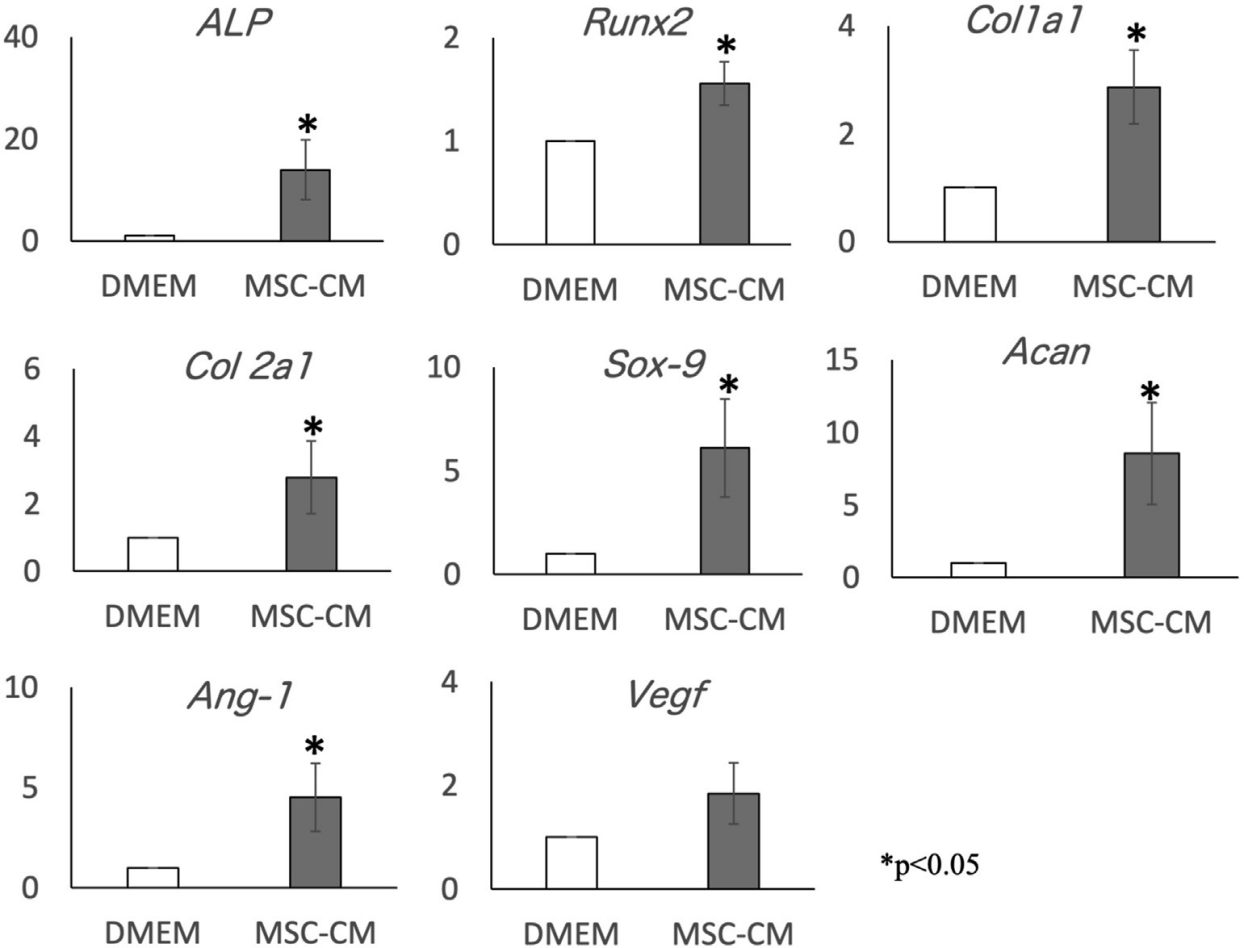
Effects of MSC-CM on rMSC gene expression. Relative expression levels of *ALP*, *Runx2*, *Col1a1*, *Col2a1*, *Sox-9*, *Acan*, *Ang-1*, and *Vegf* in rMSCs cultured with MSC-CM were analyzed (∗p < 0.05). Expression of *ALP*, *Runx2*, *Col1a1*, *Col2a1*, *Sox-9*, *Acan*, and *Ang-1* was significantly and comparably upregulated in cells exposed to MSC-CM than in cells cultured in basal DMEM, and *Vegf* also tended to be upregulated compared to that in control cells. *ALP:* alkaline phosphatase, *Runx2:* Runt-related transcription factor 2*, COL Iα:* type I alpha1 collagen, *COL 2α*: type II alpha1 collagen, *SOX9:* SRY-box transcription factor 9, *ACAN:* aggrecan, *ANG1:* angiopoietin 1, *VEGF:* vascular endothelial growth factor.

Expression of *ALP*, *Runx2*, *Col1a1*, *Col2a1*, *Sox-9*, *Acan*, and *Ang-1* was significantly and comparably upregulated in cells exposed to MSC-CM compared to cells cultured in basal DMEM; *Vegf* also tended to be upregulated in the cells exposed to MSC-CM than in control cells (Figure 3).

### 3.3 MSC-CM alleviates the reduction of bone volume and bone mineral density in CR

**Figure 4:**
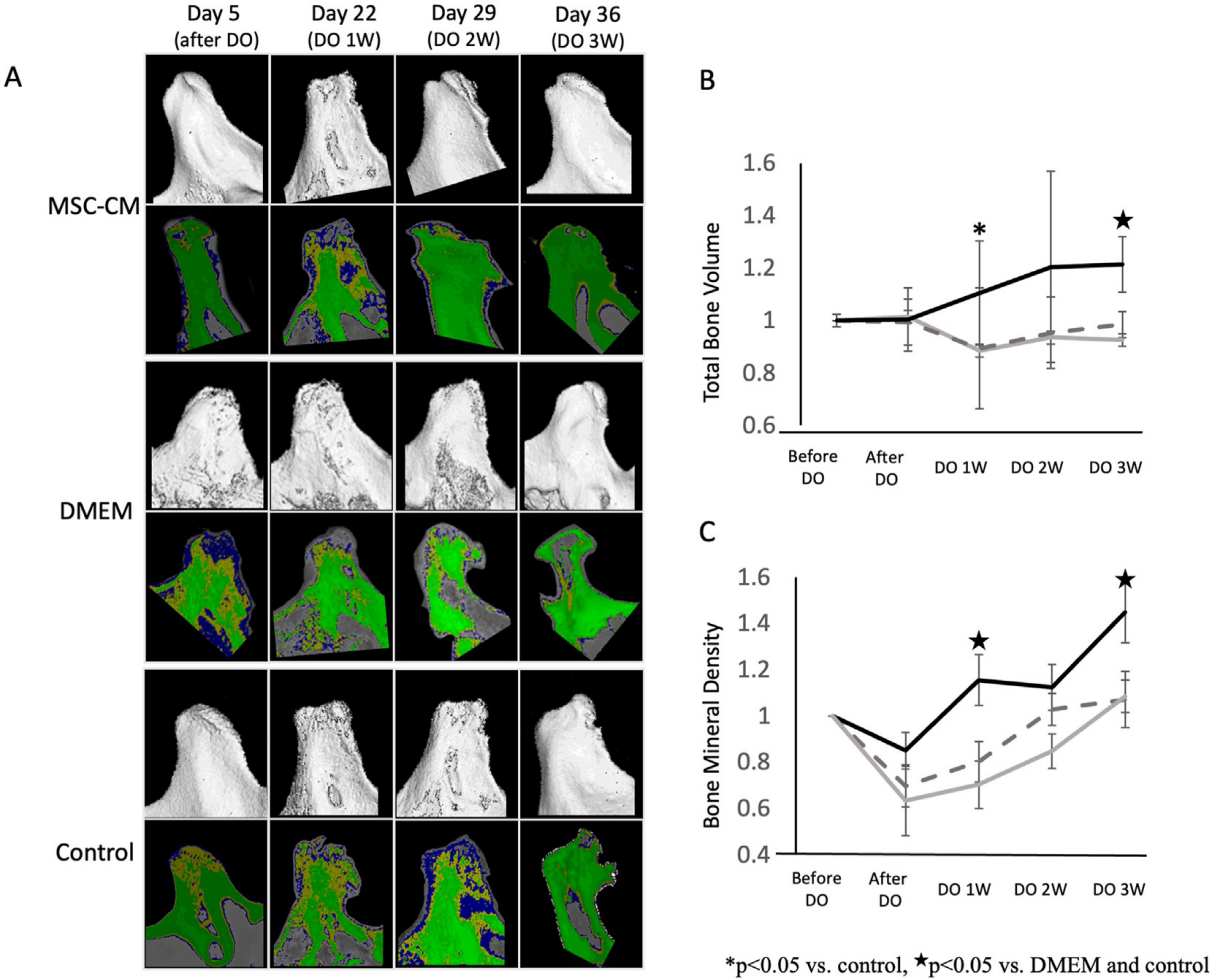
Morphological and qualitative evaluation of the condylar bone over a period of time in the same rat in three experimental groups using micro-CT. (A) In each group, images in the upper panels represent typical micro-CT images of the condylar bone and images in lower panels represent CT value-based color maps of the sliced region of condylar bone. Images were generated from the same rat evaluated over a time period (from day 5–36) in each group. CR was observed after distraction osteogenesis (DO) of the rat mandibular bone; these findings suggested that CR had occurred in this rat model. CR improved in MSC-CM‒treated group with time, although in other groups CR remained unaltered. (B) Alteration of BV with time in each experimental group. In MSC-CM group, BV improved over time and showed statistically significant improvement after 1- and 3-weeks following DO. (C) Change in BMD with time in each experimental group.BMD after completing DO was also improved over time in the MSC-CM group, especially statistically significant differences were noted after 1 and 3 weeks in this group than in both DMEM and control groups. Black line: MSC-CM, Gray dotted line: DMEM, Gray line: control, ∗p < 0.05 vs. control, ^★^p < 0.05 vs. DMEM and control.

**Figure 5:**
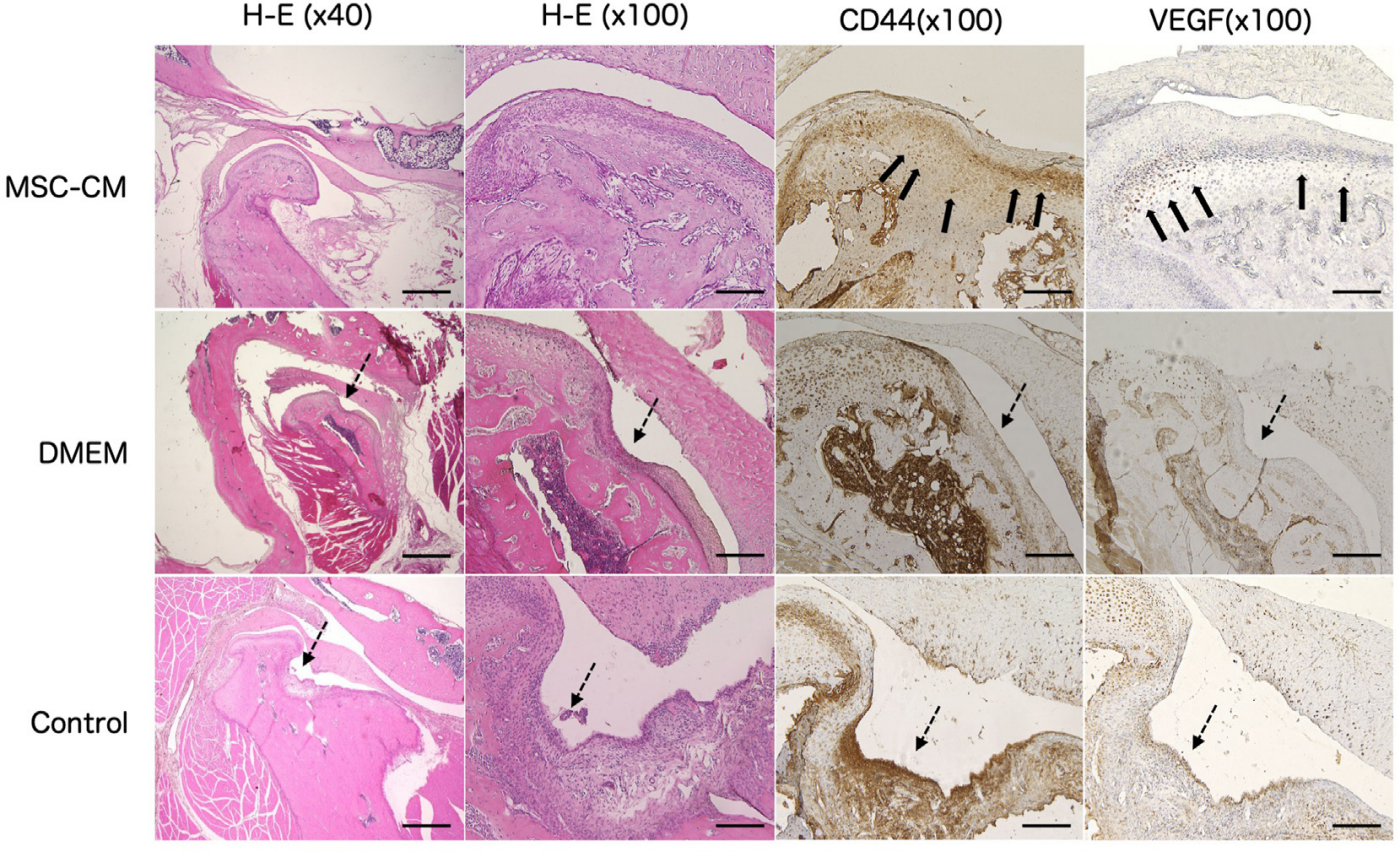
Histological and immunohistochemical evaluations. Hematoxylin and Eosin staining of the condylar specimens showed a smooth surface with a thick cartilage layer in the MSC-CM group, although CR with thinning of the cartilage layer was seen in DMEM and control groups (dotted arrows). Immunohistochemically, chondrocytes around the CR lesion showed decreased expression when treated with anti-CD44 and anti-VEGF antibodies in DMEM and control groups, whereas the thickness of the cartilage layer and expression of VEGF in the zone of hypertrophic chondrocytes as well as CD44-positive cells in the zone of proliferating chondrocytes appeared to increase in the MSC-CM group (thick arrows). Bar = 1000μm (H-E x40) and 200μM (others).

Structural changes such as erosion at the surface of the condyle and reduction in condylar height were observed after distraction osteogenesis of the rat mandibular bone. These findings suggested that CR had occurred in this rat model (Figure 4A and Figure 5).

Structural parameters of the condylar bone were investigated by calculating the BV, TV, and BMD (Figure 4B, C).

Condylar BV gradually improved in the MSC-CM group over a period of time after completion of DO and statistically improved after 3 weeks than in both the DMEM and control groups. Also, BMD was improved over time in the MSC-CM group, especially statistically significant differences were noted after 1 and 3 weeks compared with both the DMEM and control groups (Figure 4A-C).

### 3.4 MSC-CM improved bone resorption and thinning of cartilage layer of the condyle in CR

Histological findings at 3 weeks after completion of DO also revealed the occurrence of CR in the DMEM and control groups. However, in the MSC-CM group, CR improved and displayed well-ordered cell layers in cartilage and regeneration of condylar bone.

Although chondrocytes at the condyle in CR showed decreased expression when subjected to immunohistochemical staining with anti-CD44 and anti-VEGF antibodies in DMEM and control groups, thickness of the cartilage layer and expression of VEGF in the hypertrophic chondrocytes appeared to increase in MSC-CM group. Additionally, CD44-positive cells also appeared to increase in the zone of proliferating chondrocytes in MSC-CM group (Figure 5).

## 4 Discussion

Progression of CR occurs not only due to mechanical stress, but also due to condylar bone quality and volume [9, 19]. Although surgical treatment including transplantation of bone and artificial joint replacement have been proposed for the treatment of CR, these procedures only improve the height of the missing condylar bone.

In this study, we used MSC-CM to improve CR in a rat model. We have reported that MSC-CM has therapeutic effects on bone defects and medication-related osteonecrosis of the jaw [13, 14, 15, 16, 17]. MSCs secrete several secretomes (e.g., growth factors and cytokines) into conditioned medium. We identified more than 120 secretomes in a conditioned medium from human MSCs by analyzing a cytokine array [16]. Through a series of previous studies, we revealed that some of these secretomes induced cellular proliferation, cellular recruitment, osteogenesis, osteoclastogenesis, and angiogenesis [13, 14, 15, 16, 17]. We found that IGF-1, TGF-β, and VGEF were present in MSC-CM and VEGF was one of the important factors in MSC-CM that contributed toward tissue regeneration, because capillary formation could effectively recruit cells and blood to the damaged tissues [17]. In this study, immunohistochemical staining revealed that the expression of VEGF was abundant within the zone of hypertrophic chondrocytes, and thickening of the cartilage layer and condylar bone was also observed in the MSC-CM group. This phenomenon was thought to be the effect of cellular recruitment and angiogenesis by MSC-CM.

MCP-1 is known to recruit immune cells to inflamed tissues [20]. Since, monocyte chemoattractant protein (MCP)-1 was also present in MSC-CM, it was thought to contribute toward cellular recruitment.

Recently, macrophages are known to affect tissue regeneration through immunomodulatory effects induced by MSCs [21, 22]. Macrophages can switch their phenotypes using cytokines and chemokines. M1 macrophages, known as “proinflammatory macrophages”, induce inflammation, whereas M2 macrophages, known as “anti-inflammatory macrophages” suppress inflammation and contribute to tissue regeneration [23]. Inducible nitric oxide synthase (iNOS) is an intracellular marker for M1 macrophages, and MCP-1 is thought to be one of the factors that induce macrophage phenotype switching [21, 22, 23]. Chen et al. [24] reported that MSC-CM downregulated iNOS expression in lipopolysaccharide (LPS)-stimulated chondrocytes. Therefore, MSC-CM has the potential to improve CR through the switching of macrophage phenotypes and preparation of a favorable environmental for tissue regeneration.

Chen et al. [24] also reported that MSC-CM significantly downregulated the expression of inflammation-related genes such as *TNF-α, IL-1 β, and IL-6*, and upregulated extracellular matrix (ECM)-synthesis-related genes (*aggrecan*) in LPS-stimulated chondrocytes *in vitro*.

Clinically, CR is often classified as a severe form of osteoarthritis (OA), sharing some characteristics features with each other [25]. Inflammation, loss of the extracellular matrix of cartilage, and apoptosis of chondrocytes are the main pathologies of OA [26]. Recently, therapeutic effects of MSC-CM for OA have been reported [24, 27, 28, 29]. One of these studies reported that MSC-CM demonstrated therapeutic effects by protecting the microarchitecture of subchondral bone, maintaining homeostasis of matrix, and enhancing autophagy. Autophagy is a self-protective cellular mechanism against apoptosis when exposed to stress stimuli and has been proved to provide a protective mechanism in normal cartilage [29].

In this study, abundant staining with CD44 antibody was observed within the improved zone of proliferating chondrocytes in the MSC-CM group. CD44 is one of the adhesion molecules for hyaluronic acid. This indicates that MSC-CM improved the loss of ECM of the condylar tissue in this study.

Suda et al. revealed that FK506 induced more CR because of altered bone mass and architecture. Morphometric analysis of subchondral bone by micro-CT also revealed that FK506 treatment significantly reduced BV and BMD in a similar rat model as the one used in this study [9]. This suggests that CR may occur not only by mechanical stress, but also by alterations in bone mass and architecture. One of the mechanisms of MSC-CM to improve CR seemed to be the protection of microarchitecture of subchondral bone, as reported by Chen et al. [29].

Furthermore, our previous report revealed that MSC-CM showed functional maintenance of osteoclasts despite the presence of bisphosphonates, which cause apoptosis of osteoclasts [15]. Hence, it can be speculated that the other reason for improvement of CR by MSC-CM is the protection and maintenance of osteoclasts. Functional maintenance of osteoclasts also regulates the activity of osteoblasts and maintains turnover of subchondral bone, which ultimately lead to the maintenance of condylar bone mass and architecture.

Taken together these data suggest that MSC-CM has several therapeutic aspects for CR not only by enhancing cellular proliferation, cellular recruitment, osteogenesis, osteoclastogenesis, and angiogenesis, as previously reported, but also by enhancing macrophage phenotype switching, ECM homeostasis, and protection of the subchondral cartilage zone.

Currently, MSCs has been isolated from various origins including oral tissue such as dental pulp stem cells (DPSCs), human exfoliated deciduous teeth stem cells (SHED), human periodontal ligament stem cells (PDLSCs), Bone marrow stromal cells (BMSCs) and so on. DPSCs, SHEDs and PDLSCs maintain a higher growth potential in comparison to BMSCs [30]. SHED-CM was also reported to have a great potential for bone regeneration [31] and TMJ-OA [32]. In this study, we focused on the effects of MSC-CM on CR. Starting with this study, conditioned medium from various origin should be evaluated for the therapeutic effects.

By understanding the effects of conditioned medium on CR, we began to recognize that there are many phenomena occurring in the condylar tissue that have not yet been investigated. Further studies on the effects of regenerative medicine for CR are necessary to overcome this pathology.

## Declarations

### Author contribution statement

Wataru Katagiri: Conceived and designed the experiments; Performed the experiments; Analyzed and interpreted the data; Wrote the paper.

Satoshi Endo, Ryoko Takeuchi, Daisuke Suda: Performed the experiments.

Naoaki Saito, Tadaharu Kobayashi: Analyzed and interpreted the data.

### Funding statement

This work was supported by Grants-in-Aid for Scientific Research (C) from the 10.13039/501100003478Ministry of Health, Labour and Welfare of Japan (Nos. 15K112123, 18K09785, and 20K10113).

### Data availability statement

Data will be made available on request.

### Declaration of interests statement

The authors declare no conflict of interest.

### Additional information

No additional information is available for this paper.
